# Development and Validation of a Multidimensional Consumer Assessment Framework for Alternative-Ingredient Bakery and Confectionery Products: Implications for Nutrition Methodology and Sustainable Diet Research

**DOI:** 10.3390/foods15172950

**Published:** 2026-08-22

**Authors:** Anca Mihaela Dicu, Ruxandra-Cristina Marin, Călin Muntean, Radu Dumitru Moleriu, Anca Tudor, Teodora Piroș, Florentina Simona Barbu, Dana Gina Radu, Luminița Ștefania Bozdog

**Affiliations:** 1 Faculty of Food Engineering, Tourism and Environmental Protection, Aurel Vlaicu University of Arad, 310025 Arad, Romania; 2 Discipline of Pharmacology, Clinical Pharmacology and Pharmacotherapy, “Carol Davila” University of Medicine and Pharmacy, 050474 Bucharest, Romania; 3 Doctoral School of Biological and Biomedical Sciences, University of Oradea, 410087 Oradea, Romania; 4 Department III Functional Science, Discipline of Medical Informatics and Biostatistics, “Victor Babes” University of Medicine and Pharmacy, 300041 Timisoara, Romaniaatudor@umft.ro (A.T.);; 5 Centre for Economic Research and Consultancy, Faculty of Economics, Aurel Vlaicu University of Arad, 310032 Arad, Romania; 6 Faculty of Economics and Business Administration, West University of Timișoara, 300223 Timișoara, Romania

**Keywords:** consumer assessment framework, questionnaire validation, PLS-SEM, exploratory factor analysis, confirmatory factor analysis, test–retest reliability, alternative ingredients, bakery products, perceived nutritional value, sustainable diets

## Abstract

Background/Objectives: The transition toward healthier and more sustainable diets has increased the need for reformulated bakery and confectionery products formulated with alternative ingredients. However, validated instruments for assessing consumer acceptance of these products remain limited. This study aimed to develop and psychometrically validate a multidimensional assessment framework integrating food choice motives, green (environmental) values, health consciousness, perceived nutritional value, willingness to pay a premium, and purchase intention. Methods: A cross-sectional survey was conducted among 504 consumers in Romania using an online questionnaire. The instrument was developed by adapting established scales and by developing a new Perceived Nutritional Value scale. Dimensionality was evaluated using exploratory (EFA) and confirmatory (CFA) factor analyses. Temporal stability was assessed in an independent test–retest subsample (*n* = 30; 14-day interval), and the proposed conceptual model was evaluated using Partial Least Squares Structural Equation Modeling (PLS-SEM) with 5000 bootstrap resamples. Robustness was examined through a sensitivity analysis excluding respondents younger than 18 years (*N* = 441). Results: EFA (KMO = 0.933) and CFA (CFI = 0.965, TLI = 0.959, RMSEA = 0.061, SRMR = 0.033) supported the proposed six-factor structure. All constructs demonstrated excellent internal consistency (Cronbach’s α: 0.898–0.938), convergent validity (AVE: 0.790–0.889), discriminant validity (HTMT < 0.77), and good-to-excellent test–retest reliability (ICC: 0.82–0.91). Perceived nutritional value was positively and significantly associated with both willingness to pay and purchase intention, and willingness to pay partially mediated the association between perceived nutritional value and purchase intention. The structural model explained 46.1% of the variance in purchase intention; all hypothesized relationships were supported, and the findings remained stable in the sensitivity analysis. Conclusions: The proposed framework demonstrated good psychometric performance and provides a theory-informed measurement framework for assessing consumer acceptance of reformulated bakery and confectionery products. It may support future nutrition research, consumer studies, and the evaluation of food reformulation strategies aimed at promoting healthier and more sustainable dietary choices.

## 1. Introduction

Dietary patterns are increasingly recognized as key determinants of both human and planetary health. Diets characterized by excessive consumption of refined grains, free sugars, and energy-dense, nutrient-poor foods substantially contribute to the global burden of non-communicable diseases while simultaneously increasing greenhouse gas emissions, land use, and freshwater consumption associated with food production [1,2,3].

Consequently, international organizations advocate a transition toward dietary patterns that are simultaneously healthier and more environmentally sustainable, integrating nutritional quality with ecological responsibility [4].

Achieving this transition requires not only changes in agricultural production but also the reformulation of widely consumed processed foods to improve their nutritional and environmental profiles while maintaining consumer acceptance [5]. Bakery and confectionery products represent an important target for food reformulation because they are consumed regularly across all socio-demographic groups but are frequently characterized by high levels of refined flour, added sugars, and saturated fats [6]. Frequent consumption of such products has been linked to excess energy intake, overweight, type 2 diabetes, and cardiovascular disease, which reinforces the public-health rationale for improving their nutritional profile rather than relying solely on their exclusion from the diet [2,5]. At the same time, bread and bakery goods are staple foods produced and consumed in very large volumes, and the bakery and confectionery sector represents one of the largest segments of the European food industry, including in Romania; consequently, even modest nutritional improvements achieved in this product category may translate into meaningful population-level benefits [6,7,8].

Reformulation through the incorporation of whole-grain and gluten-free flours, dietary fiber, plant proteins, natural sweeteners, and healthier lipid sources offers a promising strategy for improving diet quality without fundamentally changing eating habits [7,8]. Such approaches are increasingly recognized as an integral component of sustainable food system transformation, aiming to improve the nutritional quality of frequently consumed products while minimizing disruption to established dietary behaviours. Moreover, a growing body of research has deliberately used bakery and confectionery matrices—including breads, biscuits, cookies, and chocolate—as vehicles for health-promoting ingredients such as dietary fiber, plant proteins, prebiotics, probiotics, and polyphenols, demonstrating that habitually consumed products, including sweets, can be transformed into effective carriers of nutritional benefit [6,7,8,9].

Although the technological feasibility of these reformulation strategies has been widely demonstrated, their successful implementation ultimately depends on consumer acceptance. Previous studies indicate that acceptance of healthier food products is influenced by a complex interaction among health consciousness, environmental values, perceived nutritional benefits, sensory expectations, and price considerations [10,11]. Accordingly, understanding the psychological and behavioural determinants underlying consumer acceptance has become a prerequisite for the successful market introduction and long-term adoption of reformulated food products.

In Central and Eastern Europe, including Romania, consumer research on reformulated bakery products remains limited despite their importance in everyday diets. Existing evidence suggests that consumers in this region are particularly sensitive to price and remain cautious toward sustainability-related product claims, emphasizing the need for context-specific assessment tools capable of capturing these perceptions [11,12,13]. The availability of robust measurement instruments is particularly important in this context, as cultural, economic, and dietary differences may influence consumer responses to reformulated foods and limit the applicability of instruments validated in other populations.

Despite the growing body of literature on organic, functional, plant-based, and clean-label foods [13,14] important methodological limitations remain. Many studies rely on isolated constructs, such as attitudes or willingness to pay, adapt measurement scales developed for different food categories without validating them in the new context, or focus primarily on testing structural relationships while providing only limited evidence regarding the psychometric quality of the underlying measurement instruments [15,16]. Consequently, comparisons across studies are often challenging, and the lack of standardized, context-specific instruments may compromise the reliability, reproducibility, and generalizability of research findings.

Recent methodological reviews have similarly highlighted the need for standardized, context-specific instruments capable of reliably assessing consumer acceptance of healthier and more sustainable foods across different populations and food categories. Such instruments are essential for nutrition research, dietary monitoring, product development, and evidence-based public health interventions because the validity of substantive conclusions depends directly on the quality of the measurement tools employed [17]. Moreover, rigorously validated instruments facilitate comparisons between studies, support cross-cultural adaptation, and provide a stronger methodological basis for evaluating consumer-oriented nutrition interventions over time.

A further conceptual gap concerns the mechanism through which general health- and sustainability-oriented beliefs are translated into concrete purchasing decisions. Health consciousness and environmental values are well-established determinants of food choice [18,19,20], whereas food choice motives, particularly those related to health and natural content, constitute central dimensions of consumer food choice behaviour [21].

These constructs reflect relatively stable personal orientations; however, they do not fully explain how consumers evaluate the nutritional advantages of specific reformulated products when making purchase decisions. Drawing on consumption value theory and perceived value theory [22,23], the present study introduces Perceived Nutritional Value (PNV) as a product-specific evaluative construct that captures consumers’ appraisal of the nutritional benefits offered by reformulated products.

Perceived Nutritional Value (PNV) is conceptualized as the proximal cognitive evaluation through which consumers judge whether products formulated with alternative ingredients provide genuine nutritional benefits rather than merely conveying a healthier image. In this framework, PNV represents a product-specific appraisal that bridges relatively stable personal values and health orientations with behavioural intentions. Although perceived value has been extensively investigated in consumer research, no validated construct has been specifically developed to assess perceived nutritional value in the context of alternative-ingredient bakery and confectionery products. This conceptual and methodological gap limits the ability to comprehensively investigate the determinants of consumer acceptance of reformulated foods and highlights the need for a dedicated measurement framework.

Against this background, the present study aimed to develop and psychometrically validate a multidimensional consumer assessment framework for alternative-ingredient bakery and confectionery products. The proposed framework integrates six theoretically grounded constructs—Food Choice Motives, Green Values, Health Consciousness, Perceived Nutritional Value, Willingness to Pay, and Purchase Intention—within a theory-driven structural model. The study evaluated the factorial structure, internal consistency, temporal stability, convergent and discriminant validity of the measurement instrument and examined the direct and indirect relationships among these constructs using Partial Least Squares Structural Equation Modeling (PLS-SEM). By providing a rigorously validated and transferable measurement framework, this study seeks to advance methodological approaches for assessing consumer acceptance of healthier and more sustainable bakery and confectionery products while supporting future nutrition, food reformulation, and consumer behaviour research.

## 2. Materials and Methods

### 2.1. Study Design and Participants

A cross-sectional online survey was conducted in Romania using a self-administered questionnaire developed in Google Forms (Google LLC, Mountain View, CA, USA). The survey was disseminated through social media platforms, university networks, and consumer-interest groups using a combination of convenience and snowball sampling. Data were collected between February and April 2026. Participation was voluntary and anonymous, and no personally identifiable information was collected. The introductory page described the study objectives, estimated completion time (5–8 min), data confidentiality procedures, and included an informed-consent section that had to be actively confirmed before the questionnaire could be completed. For respondents younger than 18 years, participation additionally required the prior written consent of a parent or legal guardian, obtained before questionnaire completion; the minimum age for participation was 15 years.

A total of 556 questionnaires were submitted; 52 responses were excluded prior to analysis due to incompleteness, resulting in a final analytical sample of 504 complete responses. Because all questionnaire items were mandatory, no missing data was recorded for the analyzed variables. The sample size achieved exceeded the minimum recommendations for Partial Least Squares Structural Equation Modeling (PLS-SEM) (SmartPLS version 4.1.1.8 (SmartPLS GmbH, Monheim am Rhein, Germany)). According to the ten-times rule, the minimum sample size equals ten times the largest number of structural paths directed at any single construct in the model; because no construct in the proposed model receives more than two structural paths, this criterion implies a minimum of only 20 observations [24,25]. Because the ten-times rule is widely regarded as overly liberal, the required sample size was additionally determined using the inverse square root method, the currently recommended approach for statistical power analysis in PLS-SEM assuming a minimum true standardized path coefficient of 0.20, a significance level of 5%, and a statistical power of 80%, the required sample size is approximately 155 observations. Moreover, with N = 504 the study provides 80% statistical power (α = 0.05) to detect standardized path coefficients as small as 0.111 [26]; the smallest path coefficient observed in the structural model (β = 0.218) was therefore detectable with power substantially above 80%. The sample size was therefore considered adequate for robust estimation of both the measurement and structural models.

Because the primary objective of the study was the development and psychometric validation of a measurement instrument rather than the estimation of population prevalence, the sample was not intended to be nationally representative. Consequently, the use of non-probability sampling was considered appropriate for instrument validation studies. As expected from the recruitment strategy, respondents were predominantly female, urban residents, younger adults, and highly educated. Specifically, the final sample (N = 504) comprised 70.8% women, 70.6% urban residents, and 62.0% respondents holding at least a bachelor’s degree; 12.5% of respondents were adolescents younger than 18 years (minimum age 15 years). Adolescents were eligible for inclusion because they are regular and increasingly autonomous consumers of bakery and confectionery products, and their participation—conditional on parental or legal-guardian consent—was approved by the ethics committee; the robustness of all results to the exclusion of this subgroup was verified in a dedicated sensitivity analysis (Section 3.9). Income categories refer to net monthly income expressed in Romanian lei (RON; average exchange rate during the data-collection period: EUR 1 ≈ RON 5.095; median of the daily National Bank of Romania reference rates, February–April 2026).

To evaluate the temporal stability of the instrument, an independent convenience sample completed a paper-based version of the final questionnaire on two occasions separated by a 14-day interval. Thirty participants completed both administrations and were included in the test–retest reliability analysis. This subsample was independent of the participants included in the main psychometric validation. A 14-day interval is widely recommended for test–retest studies because it minimizes recall bias while maintaining the stability of the measured constructs [27].

The study was approved by the Research Ethics Subcommittee of “Aurel Vlaicu” University of Arad (approval no. 16, date 5 February 2026). All study procedures were conducted in accordance with the Declaration of Helsinki and established principles of good biomedical research practice.

### 2.2. Instrument Development and Content Validity

#### 2.2.1. Questionnaire Development

Instrument development followed established recommendations for questionnaire construction and psychometric validation, including construct definition, item generation, adaptation of validated scales, content evaluation, translation and cultural adaptation, cognitive pretesting, and preparation of the final instrument [13,14,28]

The questionnaire was designed to assess consumer acceptance of bakery and confectionery products formulated with alternative ingredients by integrating established constructs from food choice and consumer behavior research with a newly developed Perceived Nutritional Value (PNV) construct. The overall questionnaire development and validation process is summarized in Table 1**.**

#### 2.2.2. Source Scales and Item Development

The initial item pool was developed through a targeted review of the literature and comprised both adapted and newly generated items. Green Values were assessed using items adapted from the GREEN consumption values scale [19], Health Consciousness from Gould’s Health Consciousness Scale [20], and Food Choice Motives from the health and natural-content dimensions of the Food Choice Questionnaire [21]. Willingness to Pay and Purchase Intention were adapted from previously validated instruments grounded in willingness-to-pay, perceived value, and the Theory of Planned Behaviour [19,29].

All adapted items were reformulated to specifically refer to bakery and confectionery products formulated with alternative ingredients while preserving the conceptual meaning of the original instruments.

Because no validated instrument was available to specifically assess consumers’ perceptions of the nutritional benefits of reformulated bakery and confectionery products, a novel four-item reflective Perceived Nutritional Value (PNV) scale was developed. Item generation was informed by literature on food reformulation, nutrition perception, and clean-label products [6,11,14], with the objective of capturing consumers’ cognitive evaluation of the perceived nutritional advantages associated with the use of alternative ingredients.

#### 2.2.3. Content Validity

Content validity was established through literature-based item development and expert review. The preliminary questionnaire was independently evaluated by an external expert with expertise in consumer behavior and psychometric instrument development and subsequently reviewed by the multidisciplinary research team, comprising specialists in food engineering, nutrition, and consumer economics. Items were assessed for relevance, clarity, comprehensibility, and conceptual consistency with their intended latent constructs. Based on the feedback received, minor wording refinements were introduced to improve readability and conceptual precision without modifying the underlying construct definitions or item content. Content validity was evaluated qualitatively; item-level and scale-level content validity indices (I-CVI/S-CVI) were not computed because the external expert panel comprised fewer raters than the minimum recommended for stable quantitative indices [30]. This aspect is explicitly acknowledged as a limitation in Section 4.

#### 2.2.4. Translation and Cognitive Pretesting

The questionnaire was administered in Romanian. Items adapted from English-language instruments were translated and culturally adapted by bilingual members of the research team, prioritizing conceptual rather than literal equivalence. The translated version underwent a final review by the research team to ensure linguistic clarity, cultural appropriateness, and consistency with the intended conceptual meaning before administration to the study population. Prior to full deployment, the pre-final Romanian version was cognitively pretested with a convenience sample of 10 members of the target population (adult and adolescent consumers not involved in the main survey), using a think-aloud procedure with verbal probing to evaluate item clarity, comprehension, readability, and questionnaire flow. Completion time during pretesting ranged from 5 to 8 min, no item was reported as unclear or ambiguous, and only minor wording refinements were introduced; no items were added or removed. Responses collected during cognitive pretesting were not included in the analytical dataset.

#### 2.2.5. Questionnaire Structure

All attitudinal items were measured using a five-point Likert scale ranging from 1 (strongly disagree) to 5 (strongly agree). A five-point rather than a seven-point format was adopted for the following reasons, which apply to each of the six constructs analyzed (Food Choice Motives, Green Values, Health Consciousness, Perceived Nutritional Value, Willingness to Pay, and Purchase Intention). First, using a single common response format across all constructs prevents the systematic method variance that can arise when response scales with different numbers of categories are mixed within the same instrument. Second, methodological evidence on agree-disagree scales indicates that five response categories achieve equal or higher measurement quality (reliability and validity) than seven or eleven categories, with additional categories increasing respondent burden without measurable psychometric gains [31]. Third, the five-point format reduces cognitive load in a heterogeneous general-population sample that completed the questionnaire predominantly on mobile devices and that included adolescent respondents. Fourth, the five-point format maximizes comparability with previous consumer studies on food choice and sustainable food consumption conducted in Romania and Central and Eastern Europe [12,14]. The adequacy of this choice was verified empirically: all items exhibited sufficient response dispersion (Section 3.2), and all constructs achieved excellent reliability and convergent validity (Section 3.3, Section 3.4 and Section 3.5).

The complete questionnaire comprised eleven attitudinal and behavioral domains. However, psychometric validation and structural modeling focused on six predefined reflective constructs comprising 22 items: Food Choice Motives, Green Values, Health Consciousness, Perceived Nutritional Value, Willingness to Pay, and Purchase Intention [24].

These constructs were selected *a priori* according to the proposed conceptual framework linking health- and sustainability-related values with consumers’ acceptance of bakery and confectionery products formulated with alternative ingredients.

Additional domains, including the New Environmental Paradigm, Food Neophobia, consumption frequency, trust in food labeling, and word-of-mouth communication—were collected for descriptive and exploratory analyses but were not incorporated into the structural model because they were outside the predefined theoretical framework. The complete questionnaire, including both the validated constructs and the additional exploratory domains, is provided in Supplementary Tables S1 and S2.

### 2.3. Conceptual Model and Hypotheses

The proposed conceptual model integrates six latent constructs representing antecedent dispositions, cognitive evaluations, and behavioral outcomes associated with consumer acceptance of bakery and confectionery products formulated with alternative ingredients. Food Choice Motives (FCM) and Green Values (GRV) were specified as exogenous constructs reflecting relatively stable consumer orientations. Health Consciousness (HCO) and Perceived Nutritional Value (PNV) represent cognitive evaluative mechanisms through which these orientations influence purchasing behavior, whereas Willingness to Pay (WTP) and Purchase Intention (PUI) constitute the primary behavioral outcomes. The model was grounded in consumption value theory, perceived value theory, and consumer decision-making research, which collectively suggest that personal values influence purchasing intentions indirectly through product-specific evaluations and economic considerations [19,20].

Based on the theoretical framework and previous literature, the following hypotheses were tested:

**H1.**  *Food Choice Motives positively influence Health Consciousness (FCM → HCO).*

**H2.**  *Green Values positively influence Health Consciousness (GRV → HCO).*

**H3.**  *Food Choice Motives positively influence Perceived Nutritional Value (FCM → PNV).*

**H4.**  *Green Values positively influence Perceived Nutritional Value (GRV → PNV).*

**H5.**  *Perceived Nutritional Value positively influences Willingness to Pay (PNV → WTP).*

**H6.**  *Perceived Nutritional Value positively influences Purchase Intention (PNV → PUI).*

**H7.**  *Willingness to Pay positively influences Purchase Intention (WTP → PUI).*

**H8.**  *Food Choice Motives indirectly influence Purchase Intention through Perceived Nutritional Value (FCM → PNV → PUI).*

**H9.**  *Green Values indirectly influence Purchase Intention through Perceived Nutritional Value (GRV → PNV → PUI).*

**H10.**  *Perceived Nutritional Value indirectly influences Purchase Intention through Willingness to Pay (PNV → WTP → PUI).*

The direction of the association between Food Choice Motives and Health Consciousness warrants explicit justification. Previous research has modeled health consciousness as an antecedent of health-related food choice motives, with motives mediating the association between health concern and attitudes toward healthy eating [32] reciprocal influences are, however, also plausible, because the repeated activation of health- and natural-content motives in everyday food decisions may reinforce a person’s general health awareness. Because the present data are cross-sectional, H1 and H2 are formulated as directional associations within a prediction-oriented framework rather than as claims of established causal order. Importantly, Health Consciousness occupies a terminal position in the model (no structural path originates from it), so the specification of this association does not affect the estimation of the mediation pathways (H8–H10) or the prediction of Purchase Intention. The alternative specification, in which Health Consciousness precedes Food Choice Motives [32], is examined in the Discussion and acknowledged as a limitation.

Formally, the hypothesized structural relationships correspond to the following system of linear equations, in which γ and β denote standardized path coefficients and ζ_1_–ζ_4_ denote the structural disturbance terms of the endogenous constructs:

HCO = γ_1_·FCM + γ_2_·GRV + ζ_1_ (H1, H2)

PNV = γ_3_·FCM + γ_4_·GRV + ζ_2_ (H3, H4)

WTP = β_1_·PNV + ζ_3_ (H5)

PUI = β_2_·PNV + β_3_·WTP + ζ_4_ (H6, H7)

The indirect effects specified in H8–H10 are defined as products of the corresponding path coefficients: H8 = γ_3_ × β_2_ (FCM → PNV → PUI), H9 = γ_4_ × β_2_ (GRV → PNV → PUI), and H10 = β_1_ × β_3_ (PNV → WTP → PUI).

**Figure 1:**
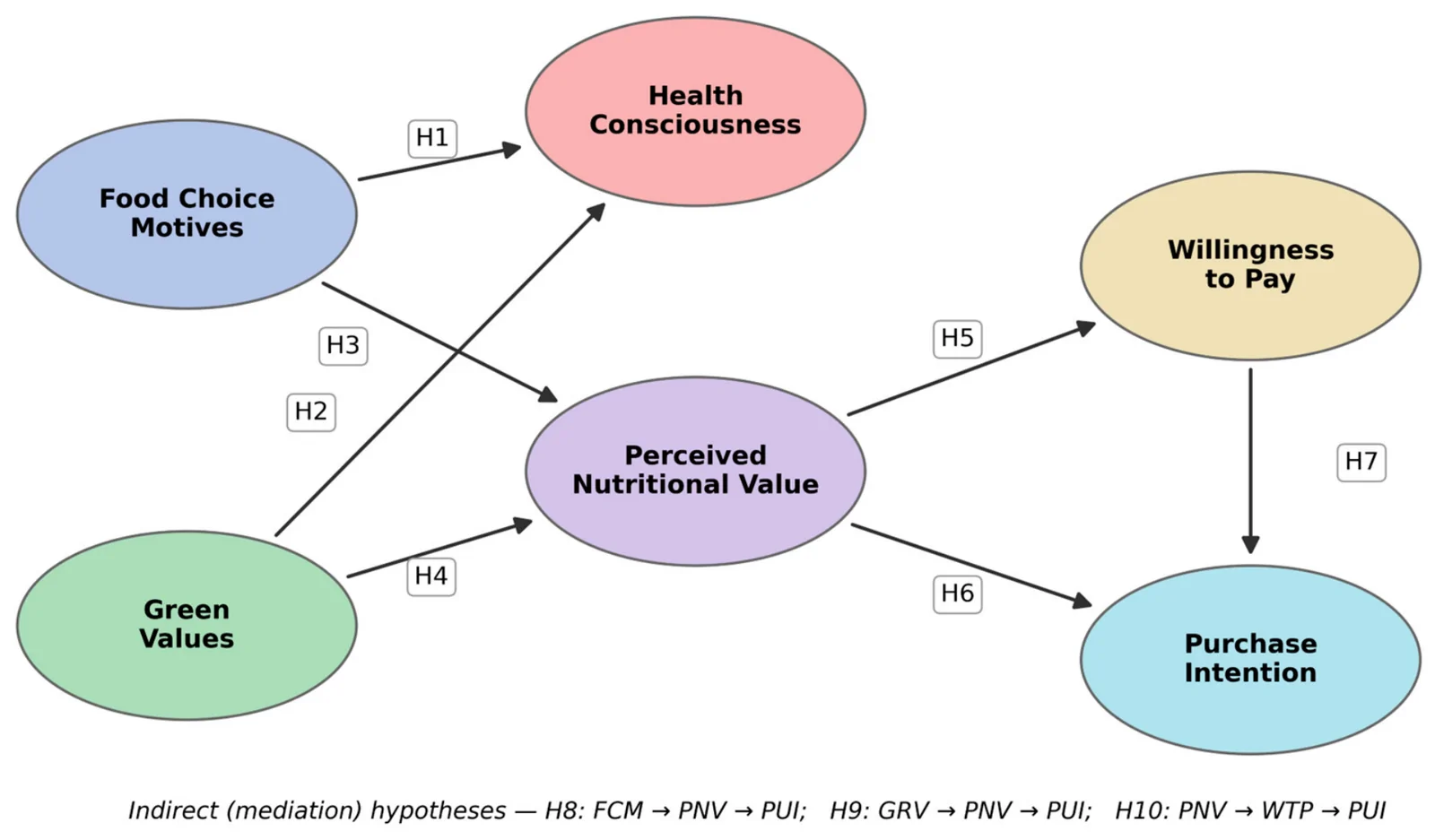
Hypothesized conceptual model linking Food Choice Motives (FCM), Green Values (GRV), Health Consciousness (HCO), Perceived Nutritional Value (PNV), Willingness to Pay (WTP), and Purchase Intention (PUI). Solid arrows represent the hypothesized direct effects (H1–H7); the indirect (mediation) hypotheses H8–H10 are defined in the text and in the equations above. Standardized path coefficients are reported in the Results section (Figure 3).

The conceptual model and hypothesized relationships are illustrated in Figure 1.

### 2.4. Statistical Analysis

All statistical analyses were performed using JASP version 0.18.3 (JASP Team, Amsterdam, The Netherlands), using R version 4.3.0 (R Foundation for Statistical Computing, Vienna, Austria) as the computational backend. Supplementary computations performed during the revision (split-half cross-validation and paired-difference analyses) were carried out in Python version 3.11 (factor-analyzer, semopy, and SciPy libraries); the equivalence of the two computational environments was verified by exactly reproducing the full-sample confirmatory factor analysis fit indices. Continuous variables were assessed for distributional normality using graphical and statistical methods. Descriptive analyses included means, standard deviations (SD), skewness, excess kurtosis, and assessment of floor and ceiling effects, with values > 15% considered indicative of notable floor or ceiling effects. Floor and ceiling effects were defined as the percentage of respondents selecting the lowest and the highest response category of each item, respectively; proportions above 15% were interpreted as notable because such concentrations of responses reduce an instrument’s discriminative capacity and may attenuate reliability and responsiveness [33] Absolute skewness values below 2 and absolute excess kurtosis values below 7 were considered acceptable for the planned factor-analytic procedures [34].

The factorial structure of the instrument was initially explored using exploratory factor analysis (EFA) based on principal-axis factoring with promax rotation. Sampling adequacy was assessed using the Kaiser–Meyer–Olkin (KMO) measure and Bartlett’s test of sphericity. The number of retained factors was determined using the Kaiser criterion (eigenvalues 1), visual inspection of the scree plot, and parallel analysis. Subsequently, confirmatory factor analysis (CFA) was performed using maximum likelihood estimation. Model fit was evaluated using the Comparative Fit Index (CFI ≥ 0.95), Tucker–Lewis Index (TLI ≥ 0.95), Root Mean Square Error of Approximation (RMSEA ≤ 0.08), and Standardized Root Mean Square Residual (SRMR ≤ 0.08), according to commonly recommended criteria [35].

PLS-SEM was selected because the primary objective of the study was the validation of a newly developed measurement instrument and the evaluation of a prediction-oriented conceptual model comprising multiple mediating relationships. In addition, the observed deviations from multivariate normality supported the use of this variance-based estimation approach [36].

The measurement model was evaluated by examining indicator loadings (≥0.708), internal consistency reliability (Cronbach’s α and composite reliability ≥ 0.70), convergent validity (average variance extracted, AVE ≥ 0.50), and discriminant validity using the Fornell–Larcker criterion and the heterotrait–monotrait ratio (HTMT < 0.85). The structural model was assessed by examining collinearity (variance inflation factor, VIF < 5), standardized path coefficients, coefficients of determination (R^2^), effect sizes (f^2^), and predictive relevance (Q^2^) obtained using the blindfolding procedure. Statistical significance of direct and indirect effects was evaluated using bias-corrected bootstrapping with 5000 resamples and 95% confidence intervals. Overall model fit was assessed using the Standardized Root Mean Square Residual (SRMR) and the Normed Fit Index (NFI), while common method bias was evaluated using the full-collinearity assessment approach [37]. Effect sizes f^2^ were interpreted as small (≥0.02), medium (≥0.15), or large (≥0.35) [36].

Temporal stability was assessed using the two-way mixed-effects intraclass correlation coefficient (ICC(2,1)) with absolute agreement and interpreted according to established recommendations as poor (<0.50), moderate (0.50–0.75), good (0.75–0.90), or excellent (>0.90) [27]. In addition, potential systematic measurement drift between the two administrations was examined using paired mean differences with 95% confidence intervals; the normality of the paired differences was first assessed using Shapiro–Wilk tests and, because significant deviations from normality were detected, the absence of systematic differences was additionally tested using Wilcoxon signed-rank tests.

Robustness was examined through a pre-specified sensitivity analysis in which the measurement and structural models were re-estimated after excluding respondents younger than 18 years (N = 441), and through an exploratory comparison of construct scores across self-reported dietary patterns (Kruskal–Wallis tests). In addition, to address the risk of overly optimistic fit estimates arising from conducting exploratory and confirmatory factor analyses on the same observations, the sample was randomly divided into two non-overlapping subsamples of equal size (n = 252 each) using a computer-generated random permutation with a fixed seed to ensure reproducibility: EFA was repeated in the first subsample and CFA in the second, independent subsample (Section 3.3).

## 3. Results

### 3.1. Sample Characteristics

The socio-demographic characteristics of the study sample are summarized in Table 2**.** A total of 504 respondents participated in the study. The sample was predominantly female (70.8%), urban (70.6%), and highly educated, with 62.0% of participants holding at least a bachelor’s degree. Most respondents reported an omnivorous diet (76.2%), whereas flexitarian (15.7%), gluten-free (4.0%), vegetarian (3.2%), and vegan (1.0%) dietary patterns were less frequently represented. Participants covered a broad age range and diverse income categories, providing sufficient variability in socio-demographic characteristics and dietary habits to support the psychometric validation of the proposed multidimensional assessment framework.

### 3.2. Item Distributions, Floor and Ceiling Effects

Descriptive statistics and distributional characteristics of all questionnaire items are presented in Table 3. Overall, item responses demonstrated adequate variability across the five-point Likert scale, with mean scores generally exceeding the scale midpoint, indicating a favorable orientation toward bakery and confectionery products formulated with alternative ingredients. Most items exhibited mild negative skewness, reflecting a tendency toward agreement, whereas excess kurtosis values remained within acceptable ranges for psychometric analyses.

No substantial floor effects were observed, indicating that responses were not concentrated in the lowest response category. Ceiling effects were more evident, particularly for items assessing Health Consciousness and Food Choice Motives, consistent with stronger endorsement of health- and nutrition-related attitudes among respondents. Nevertheless, all items demonstrated sufficient response dispersion to support subsequent exploratory and confirmatory factor analyses, as well as PLS-SEM measurement model evaluation.

### 3.3. Factorial Validity, Reliability, and Convergent Validity

Sampling adequacy was excellent (KMO = 0.933), and Bartlett’s test of sphericity was statistically significant (χ^2^ = 10,348.8, *p* < 0.001), confirming the suitability of the dataset for factor analysis. Exploratory Factor Analysis (EFA) fully supported the hypothesized six-factor structure, with all items loading strongly on their intended constructs and no meaningful cross-loadings. The extracted factors explained 80.8% of the total variance, providing strong evidence for the multidimensional structure of the proposed instrument. In the random split-half validation, EFA in the first subsample (n = 252; KMO = 0.922; Bartlett’s test *p* < 0.001) reproduced the six-factor structure, with all 22 items loading on their intended factors (all primary loadings ≥ 0.63, all cross-loadings ≤ 0.33) and the six factors jointly explaining 79.0% of the total variance, and CFA in the second, independent subsample (n = 252) confirmed an acceptable fit of the six-factor model (χ^2^/df = 2.49; CFI = 0.948; TLI = 0.939; RMSEA = 0.077; SRMR = 0.041), supporting the stability of the factorial structure beyond the sample in which it was identified.

**Figure 2:**
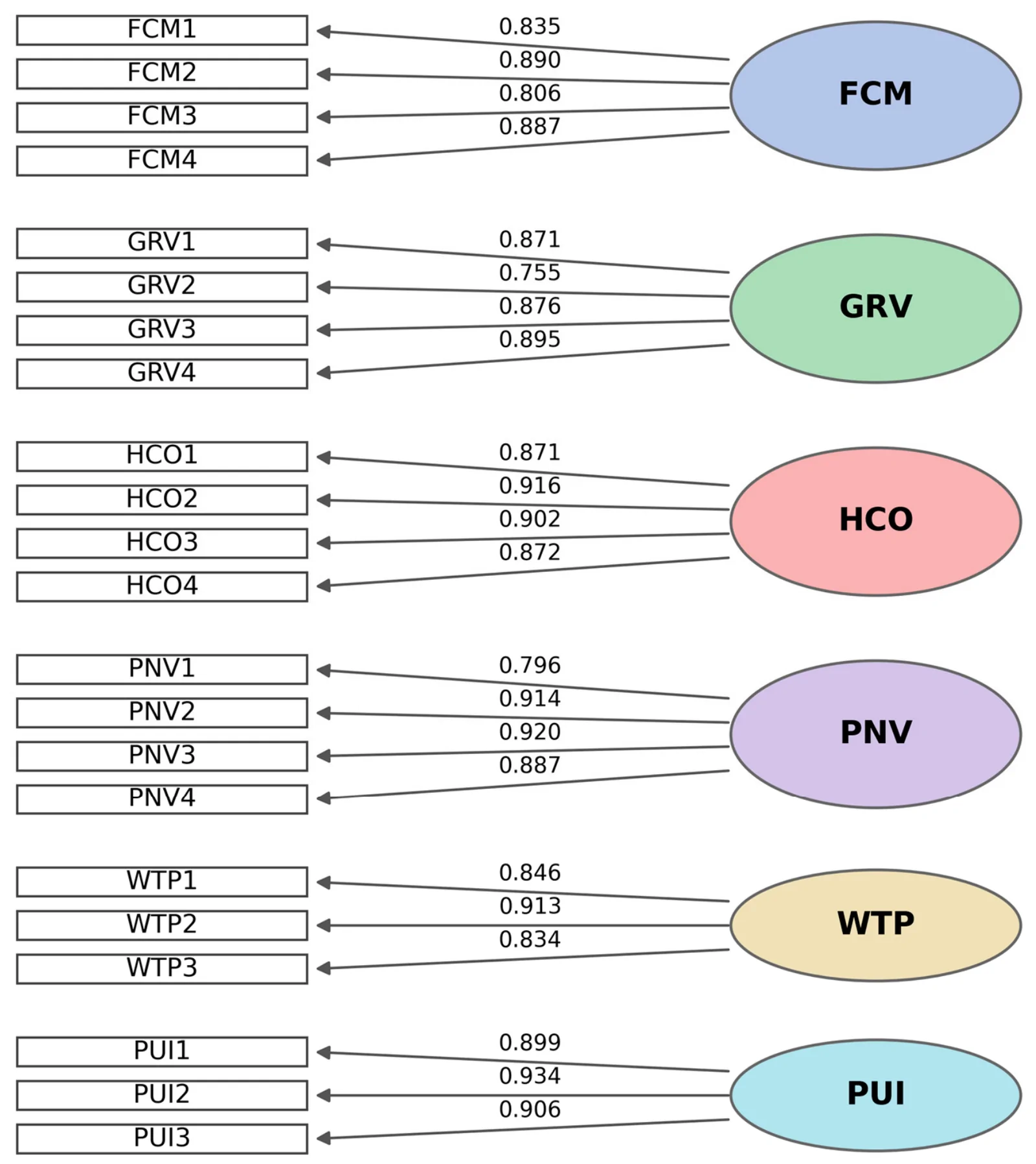
Confirmatory factor analysis: six-factor measurement model with standardized loadings (N = 504).

Confirmatory Factor Analysis (CFA) further supported the measurement model, demonstrating good-to-excellent model fit (χ^2^/df = 2.84, CFI = 0.965, TLI = 0.959, RMSEA = 0.061, SRMR = 0.033). The final six-factor CFA measurement model is presented in Figure 2. Standardized factor loadings were uniformly high, confirming that all items adequately represented their respective latent constructs (Table 4).

### 3.4. Reliability and Convergent Validity

The measurement model demonstrated excellent internal consistency reliability and convergent validity (Table 4). Cronbach’s α coefficients ranged from 0.898 to 0.938, and composite reliability values ranged from 0.936 to 0.960, exceeding the recommended threshold of 0.70 for all constructs. Likewise, average variance extracted (AVE) values ranged from 0.790 to 0.889, substantially exceeding the recommended minimum value of 0.50 and confirming satisfactory convergent validity. Collectively, these findings support the reliability and convergent validity of the proposed multidimensional consumer assessment framework.

### 3.5. Discriminant Validity

Discriminant validity was confirmed using both the Fornell–Larcker criterion and the heterotrait–monotrait ratio (HTMT) (Table 5). For all constructs, the square root of the average variance extracted (AVE) exceeded the corresponding inter-construct correlations, satisfying the Fornell–Larcker criterion. Likewise, all HTMT values were below the recommended threshold of 0.85, indicating adequate conceptual distinctiveness among the six latent constructs. Collectively, these findings provide strong evidence that each construct measured a unique aspect of consumer acceptance while maintaining satisfactory discriminant validity.

### 3.6. Test–Retest Reliability (Temporal Stability)

The proposed instrument demonstrated good temporal stability over the 14-day test–retest interval (Table 6). Intraclass correlation coefficients (ICC(2,1)) ranged from 0.823 to 0.909, indicating good-to-excellent reliability across all six constructs. The newly developed Perceived Nutritional Value construct exhibited the highest temporal stability (ICC = 0.909), whereas all remaining constructs also demonstrated good stability over time (ICC ≥ 0.823). Mean construct scores were similar at the two administrations; paired mean differences (T2 − T1) were small for all constructs: Food Choice Motives −0.083 (95% CI −0.225 to 0.058), Green Values 0.058 (−0.104 to 0.220), Health Consciousness 0.000 (−0.175 to 0.175), Perceived Nutritional Value 0.100 (−0.024 to 0.224), Willingness to Pay 0.022 (−0.147 to 0.192), and Purchase Intention 0.044 (−0.143 to 0.231) points on the five-point scale, and none of the 95% confidence intervals excluded zero. Because Shapiro–Wilk tests indicated that the paired differences deviated from normality (all *p* < 0.05), the absence of systematic differences was verified using Wilcoxon signed-rank tests, none of which was statistically significant (all *p* ≥ 0.119), indicating no evidence of systematic measurement drift.

### 3.7. Structural Model Assessment and Hypothesis Testing

Collinearity diagnostics indicated no multicollinearity concerns among predictor constructs, with all inner variance inflation factor (VIF) values below the recommended threshold. The structural model demonstrated satisfactory explanatory power and predictive relevance, accounting for a substantial proportion of variance in the endogenous constructs (HCO: R^2^ = 0.572; PNV: R^2^ = 0.361; WTP: R^2^ = 0.302; PUI: R^2^ = 0.461). Positive Q^2^ values for all endogenous constructs further confirmed the model’s predictive capability.

**Figure 3:**
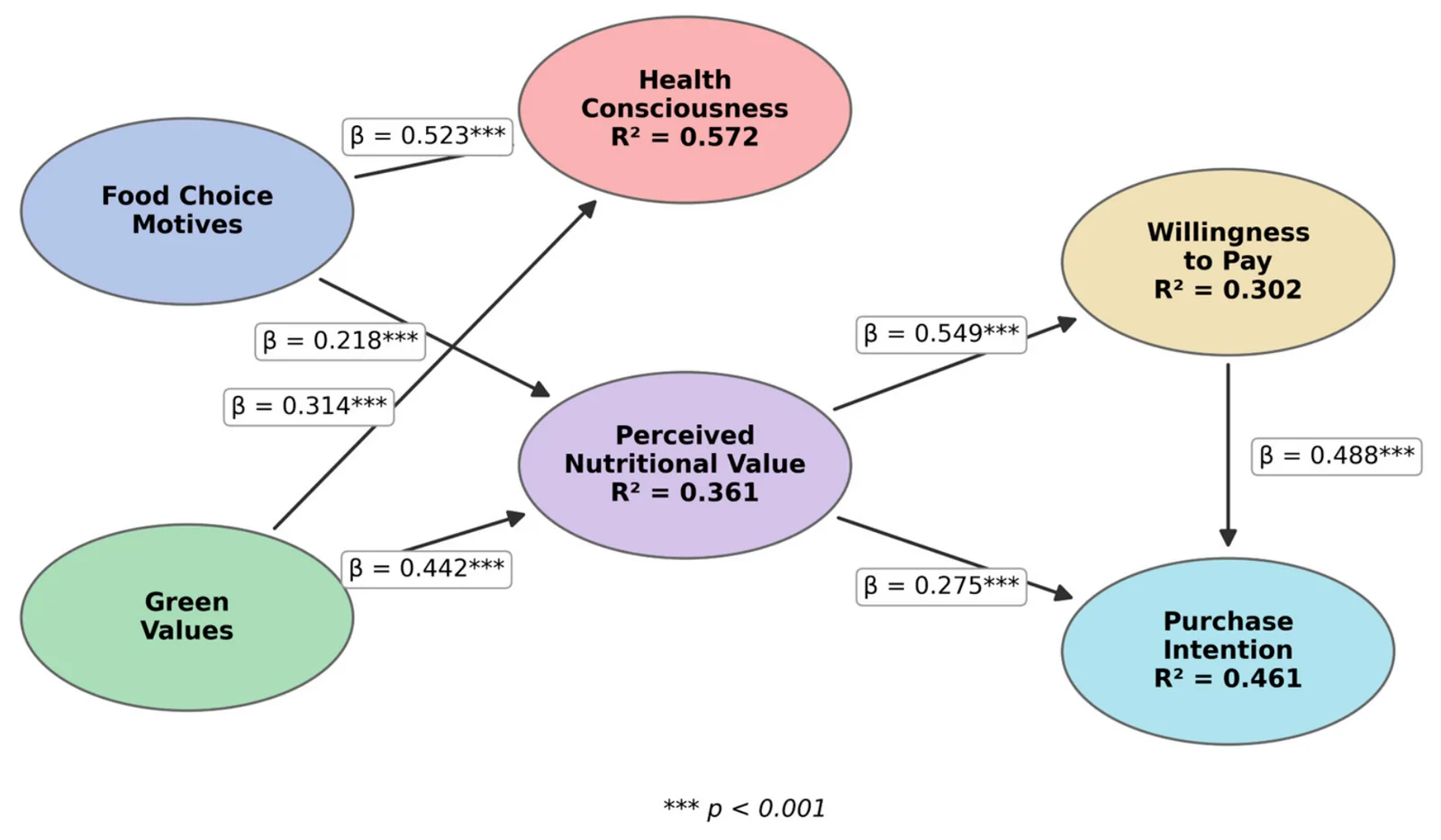
Validated structural model with standardized path coefficients (N = 504; 5000-resample bootstrap). R^2^ is shown for each endogenous construct. *** *p* < 0.001.

The estimated path coefficients and hypothesis-testing results are summarized in Table 7. All seven hypothesized direct relationships (H1–H7) were statistically supported. Food Choice Motives exerted the strongest direct effect on Health Consciousness (β = 0.523), whereas Green Values showed the strongest direct influence on Perceived Nutritional Value (β = 0.442). Perceived Nutritional Value emerged as the central cognitive construct within the proposed framework, exerting significant positive effects on both Willingness to Pay (β = 0.549) and Purchase Intention (β = 0.275). In turn, Willingness to Pay was a strong predictor of Purchase Intention (β = 0.488), supporting the proposed behavioral pathway linking consumers’ perceptions of nutritional value to purchasing intentions. The validated structural model with standardized path coefficients and R^2^ values is presented in Figure 3.

### 3.8. Mediation Analysis

The mediation analysis supported all three hypothesized indirect effects (Table 8). Perceived Nutritional Value significantly mediated the associations of both Food Choice Motives and Green Values with Purchase Intention, indicating that consumers’ evaluation of the nutritional benefits of alternative-ingredient bakery and confectionery products represents a key cognitive mechanism linking individual values and food-choice motivations with purchasing intentions. Both mediation pathways were classified as indirect-only, indicating that the effects of Food Choice Motives and Green Values on Purchase Intention were transmitted exclusively through Perceived Nutritional Value.

Willingness to Pay also significantly mediated the relationship between Perceived Nutritional Value and Purchase Intention. This complementary mediation suggests that consumers’ perceptions of nutritional value influence purchasing intentions both directly and indirectly through an increased willingness to pay, supporting the sequential behavioral pathway proposed in the conceptual model.

### 3.9. Robustness: Sensitivity Analysis and Diet-Type Comparison

The robustness analysis confirmed the stability of the proposed measurement framework (Table 9). Re-estimation of the structural model after excluding respondents younger than 18 years (N = 441) yielded results that were virtually identical to those obtained for the full sample. Internal consistency reliability and convergent validity remained essentially unchanged, all structural relationships retained statistical significance, and all hypothesized associations were supported, indicating that the inclusion of younger respondents did not materially influence the psychometric properties or structural relationships of the proposed framework.

An exploratory comparison of construct scores across self-reported dietary patterns (omnivore, flexitarian, gluten-free, vegetarian, and vegan) revealed no statistically significant differences (Kruskal–Wallis, all *p* ≥ 0.087). These findings suggest that the proposed measurement framework demonstrates consistent performance across diverse dietary groups, further supporting its robustness and potential applicability in heterogeneous consumer populations.

Overall, the full model demonstrated acceptable fit (SRMR = 0.077; NFI = 0.899). Collectively, the psychometric analyses provided consistent evidence supporting the reliability, validity, and robustness of the proposed measurement framework.

## 4. Discussion

This study developed and psychometrically validated a multidimensional framework for assessing consumer acceptance of bakery and confectionery products formulated with alternative ingredients. The findings demonstrate that the proposed instrument possesses a stable factorial structure, excellent reliability, convergent and discriminant validity, and good temporal stability. The consistency of these findings across multiple complementary psychometric approaches, including exploratory and confirmatory factor analyses, PLS-SEM, test–retest reliability, and sensitivity analyses, supports the robustness of the proposed measurement framework. Beyond instrument validation, the structural model provided empirical support for all hypothesized relationships and explained a substantial proportion of the variance in purchase intention. Together, these results indicate that the proposed framework can provide a reliable basis for investigating consumer acceptance of reformulated bakery and confectionery products and may facilitate future research examining the factors influencing healthier and more sustainable food choices.

The most important finding of this study is the central role of PNV within the proposed conceptual model. Rather than representing only an evaluation of product quality, PNV emerged as the key cognitive construct linking consumers’ health- and sustainability-related orientations with purchase intention. Although Food Choice Motives and Green Values were associated with downstream behavioral outcomes, their effects were transmitted through consumers’ perceptions of the nutritional value of products formulated with alternative ingredients. These findings suggest that consumers are more likely to express purchase intentions when they perceive that reformulated bakery and confectionery products offer meaningful nutritional benefits rather than simply conveying a healthier image. This interpretation is consistent with consumption-value and perceived-value theories [22,23,29,38] and aligns with recent evidence indicating that perceived value represents one of the strongest determinants of consumer acceptance of healthy and sustainable food products, frequently outweighing socio-demographic characteristics or general environmental attitudes [39]. Our findings extend this evidence by showing that, in the context of reformulated bakery and confectionery products, perceived nutritional value occupies a central position within the proposed conceptual framework and is closely associated with both willingness to pay and purchase intention.

Each construct in the proposed framework is anchored in an established theoretical tradition. Food Choice Motives derive from the motivational framework underlying the Food Choice Questionnaire, which conceptualizes food selection as driven by a finite set of motives, including health and natural content [21,40]. Green Values reflect green consumption values theory [18] and are consistent with value-based accounts of environmentally significant behavior, such as the value-belief-norm theory [40] Health Consciousness corresponds to the enduring individual disposition described by Gould [19] and is conceptually related to the individual-readiness constructs of the Health Belief Model [41] Perceived Nutritional Value is grounded in consumption value theory [38] and perceived value theory [22,23], which posit that purchase decisions depend on consumers’ overall assessment of the benefits obtained relative to what is sacrificed. Willingness to Pay reflects the price-value trade-off that is central to perceived value theory [22,23], whereas Purchase Intention is conceptualized in accordance with the Theory of Planned Behaviour, in which intention constitutes the most proximal determinant of behavior [29]. Together, these complementary theoretical perspectives provide the rationale for the value → evaluation → intention sequence tested in the structural model.

The newly developed PNV scale represents an important methodological contribution of the present study. Although previous research has examined perceived value in relation to organic, functional, and clean-label foods [11,14,15,42], no validated instrument specifically measuring consumers’ perceptions of the nutritional value of reformulated bakery and confectionery products has been available. The proposed scale demonstrated excellent internal consistency, convergent and discriminant validity, and good temporal stability, supporting its ability to consistently capture this construct within the study population. These findings suggest that perceived nutritional value can be assessed as a distinct dimension of consumer evaluation rather than being inferred from broader measures of perceived value or health attitudes. Although additional validation in other populations and food categories is warranted, the proposed scale provides a useful foundation for future research investigating consumer perceptions of reformulated and healthier food products.

The findings also clarify the role of WTP within the proposed conceptual framework. Perceived Nutritional Value exerted both a direct effect on Purchase Intention and an indirect effect through Willingness to Pay, indicating that consumers’ evaluations of nutritional value influence purchasing decisions through complementary pathways. Consumers who perceived greater nutritional benefits in bakery and confectionery products formulated with alternative ingredients were also more willing to accept a price premium, which in turn was associated with stronger purchase intention. However, the direct association between Perceived Nutritional Value and Purchase Intention remained significant, suggesting that consumers’ nutritional evaluations influence purchasing decisions beyond economic considerations alone. This observation is consistent with previous research highlighting that consumers are generally willing to pay a premium for healthier foods only when nutritional improvements are perceived as credible, relevant, and personally beneficial [22]. More recent studies similarly report that perceived value and perceived health benefits represent stronger predictors of willingness to pay than sustainability claims alone, particularly for reformulated and functional food products [39].

The relationships among the antecedent constructs further improve the understanding of consumer acceptance of reformulated foods. Food Choice Motives showed the strongest association with Health Consciousness, whereas Green Values were more strongly associated with Perceived Nutritional Value. These findings suggest that health-related food motives and environmental values contribute to consumer acceptance through partially different pathways within the proposed framework. Consumers primarily motivated by health tended to report greater health consciousness, whereas those with stronger environmental values were more likely to perceive additional nutritional benefits in products formulated with alternative ingredients. This interpretation is consistent with recent evidence showing that health-related behaviors are embedded within broader lifestyle patterns, where individual values, awareness, and daily habits interact to shape health-oriented decisions and behavioral transitions [43]. Similar relationships have been reported for sustainable, plant-based, and clean-label foods, where environmental attributes frequently generate positive expectations regarding healthfulness and overall product quality [10,11,14].

An important consideration concerns the direction of the association between Food Choice Motives and Health Consciousness. Consistent with evidence that health concern shapes food choice motives, previous research has modeled health consciousness as an antecedent of health-related motives, with motives mediating the association between health concern and attitudes toward healthy eating [32] Given the cross-sectional design of the present study, the two specifications cannot be empirically distinguished, and the estimated FCM → HCO path should therefore be interpreted as a directional association rather than a causal effect. Importantly, because Health Consciousness does not transmit effects to any downstream construct in the proposed model, re-specifying the direction of this single association leaves the estimation of the perceived nutritional value and willingness-to-pay pathways (H3–H10), and hence all substantive conclusions regarding purchase intention, unchanged. Longitudinal or experimental designs are needed to clarify the causal ordering of these dispositional constructs.

Recent studies also indicate that consumers tend to perceive sustainable alternatives as a distinct product category whose acceptance depends largely on perceived healthiness, naturalness, and trust rather than on environmental benefits alone [44]. Taken together, these findings support the view that consumers evaluate reformulated foods by integrating multiple personal values and product-related perceptions rather than relying on a single decision criterion.

The present findings also have important methodological implications for nutrition research. Although consumer acceptance of organic, functional, plant-based, and clean-label foods has been extensively investigated, relatively few studies have focused on the rigorous psychometric evaluation of the instruments used to measure these constructs. Existing research frequently adapts questionnaires developed for different food categories without systematically evaluating their factorial structure, reliability, validity, and temporal stability in the target context. This may limit the comparability of findings across studies and reduce confidence in the measurement of consumer-related outcomes. By integrating well-established constructs with a newly developed PNV scale and evaluating the complete framework using complementary psychometric approaches, including exploratory and confirmatory factor analyses, PLS-SEM, test–retest reliability, and sensitivity analyses [24,28,36,45,46], the present study provides a comprehensively validated measurement framework for assessing consumer acceptance of reformulated bakery and confectionery products. The consistent findings obtained across these complementary validation procedures increase confidence that the instrument measures the intended constructs in a reliable and coherent manner. This contribution is particularly relevant given recent calls for standardized and context-specific methodological tools capable of evaluating consumer acceptance of novel and reformulated foods while ensuring robust psychometric performance [47].

The practical implications of these findings extend beyond instrument validation. The structural model suggests that consumers are more likely to accept reformulated bakery and confectionery products when nutritional improvements are perceived as tangible, credible, and personally relevant. Consequently, successful product reformulation should prioritize demonstrable nutritional improvements (higher fiber content, improved protein quality, reduced sugar content, or healthier lipid profiles) supported by transparent and evidence-based communication rather than relying predominantly on environmental claims [6,8,11,42].

These findings are consistent with the observed relationships within the proposed framework, where PNV showed direct associations with both Willingness to Pay and Purchase Intention. Consumers appeared willing to consider a price premium when nutritional improvements were perceived as meaningful, suggesting that perceived nutritional value may help explain why some reformulated products are accepted despite higher prices. This interpretation is consistent with recent research showing that consumer acceptance of reformulated and plant-based foods depends primarily on perceived health benefits, ingredient transparency, and product credibility, whereas sustainability attributes alone rarely determine purchase decisions. Studies published during the past two years similarly conclude that simplified ingredient lists, recognizable ingredients, and clearly communicated nutritional benefits strengthen both consumer trust and purchase intention [48].

From a product development perspective, these findings suggest that communication strategies emphasizing scientifically supported nutritional improvements may be more effective than messages focusing exclusively on sustainability. At the same time, the results do not diminish the importance of environmental attributes but rather indicate that these attributes may be more influential when accompanied by clear nutritional benefits. This interpretation is also consistent with previous evidence showing that consumers assign greater value to products perceived as healthier, more natural, and nutritionally superior than to products promoted solely on the basis of environmental characteristics [1,2,5,22].

From a broader nutrition perspective, the present findings support current international efforts promoting food reformulation as a strategy for improving dietary quality while maintaining consumer acceptance [1,2,5].

Recent reviews emphasize that reformulation should not be viewed solely as a technological process but as part of a broader transformation of food systems in which nutritional quality, sustainability, affordability, and consumer perception interact continuously. Within this context, consumer acceptance becomes an essential component of successful reformulation because nutritionally improved products can contribute to healthier dietary patterns only if they are accepted and purchased by consumers. The proposed framework may therefore provide a useful tool for evaluating how consumers perceive reformulated bakery and confectionery products and for identifying the factors most strongly associated with their acceptance. Such information may support the design and evaluation of reformulation strategies, communication approaches, and nutrition interventions aimed at encouraging healthier food choices. Our findings therefore complement recent recommendations advocating evidence-based nutrition communication and scientifically substantiated product information to facilitate healthier and more sustainable food choices [49].

Despite these contributions, several limitations should be considered when interpreting the findings. The study employed a convenience and snowball sampling strategy, resulting in a sample with a higher proportion of female, urban, younger, and highly educated respondents. Although such sampling approaches are commonly used in the development and initial validation of psychometric instruments because they provide adequate variability for evaluating measurement properties, the findings may not be fully generalizable to the broader Romanian population. Consequently, additional validation in larger and more demographically diverse samples is warranted. Expanding validation across different socio-economic groups, geographical regions, and cultural settings would provide further evidence regarding the stability and applicability of the proposed framework.

The cross-sectional design represents another limitation, as consumer perceptions and acceptance of reformulated foods may evolve over time with increasing product availability, market exposure, and changes in dietary habits. Accordingly, the observed relationships should be interpreted as associations rather than causal effects. Longitudinal studies would therefore be valuable for examining the temporal evolution of the proposed conceptual model. Although the newly developed PNV scale demonstrated excellent psychometric performance in the present study, further validation in independent populations and across different food categories is needed to confirm its broader applicability [47].

Additional methodological limitations concern the validation strategy itself. Exploratory and confirmatory factor analyses, as well as the structural model, were estimated on the same sample of 504 respondents; although the factorial structure was corroborated in a random split-half analysis (Section 3.3), the use of a single dataset for scale development, confirmation, and structural modeling may yield somewhat optimistic estimates of model fit, and replication in fully independent samples remains necessary. Content validity was established qualitatively through expert review and was not quantified using item-level or scale-level content validity indices [30] and cognitive pretesting was conducted with a small convenience sample. Furthermore, the direction of the association between Food Choice Motives and Health Consciousness could not be established empirically, and the alternative specification in which health consciousness precedes food choice motives [40] is equally compatible with the data. Finally, the test–retest subsample was small (n = 30), so the estimates of temporal stability should be interpreted with appropriate caution.

Future research should evaluate the proposed framework in other categories of reformulated and sustainable foods, including dairy products, meat alternatives, plant-based products, and beverages. Further validation in different populations and cultural settings would help determine the generalizability of the measurement framework and the newly developed PNV construct. Additional studies combining this consumer-assessment framework with objective nutritional characteristics, sensory evaluation, front-of-pack labeling, purchasing experiments, or real-world consumer behavior could provide a more comprehensive understanding of how consumers translate perceived nutritional value into food choices. Such research may also help identify which product characteristics and communication strategies are most effective in promoting acceptance of healthier reformulated foods. Ultimately, the availability of rigorously validated, context-specific measurement instruments may facilitate the evaluation of consumer-oriented reformulation strategies and support evidence-based nutrition research aimed at encouraging healthier and more sustainable dietary patterns [1,4,18].

## 5. Conclusions

This study developed and psychometrically validated a multidimensional framework for assessing consumer acceptance of bakery and confectionery products formulated with alternative ingredients. The proposed instrument demonstrated a stable factorial structure, excellent reliability, convergent and discriminant validity, and good temporal stability, supporting its use as a reliable measurement framework for nutrition and consumer-behavior research. By integrating established determinants of food choice with the newly developed Perceived Nutritional Value construct, the framework provides a structured approach for investigating the factors associated with consumer acceptance of reformulated food products.

The findings highlight the important role of perceived nutritional value within the proposed conceptual framework. Consumers who perceived greater nutritional benefits were more likely to express purchase intention and a greater willingness to pay for products formulated with alternative ingredients, underscoring the relevance of perceived nutritional value in consumer decision-making. These findings suggest that successful food reformulation strategies should combine measurable nutritional improvements with clear, transparent, and evidence-based communication to strengthen consumer acceptance.

Although further validation in different populations and food categories is warranted, the proposed framework provides a useful methodological foundation for future studies investigating reformulated, functional, and sustainable foods. Its application may support the evaluation of consumer responses to food reformulation strategies and contribute to the development of nutrition interventions and communication approaches aimed at promoting healthier and more sustainable dietary choices.

## Figures and Tables

**Table 1:** Questionnaire development and psychometric evaluation workflow.

Stage	Description
1. Conceptual framework	Six theoretically grounded constructs were identified based on literature on food choice, perceived value, sustainability, and consumer behavior.
2. Item generation	Items were adapted from validated instruments assessing Green Values, Health Consciousness, Food Choice Motives, Willingness to Pay, and Purchase Intention. A novel Perceived Nutritional Value (PNV) scale was developed based on literature on food reformulation, nutrition perception, and clean-label products.
3. Content validity assessment	Items were reviewed by an independent expert in consumer behavior and psychometric instrument development and subsequently evaluated by the multidisciplinary research team with expertise in food engineering, nutrition, and consumer economics.
4. Translation and cognitive pretesting	English-language items were translated and culturally adapted into Romanian and subsequently cognitively pretested to assess clarity, comprehension, readability, and questionnaire flow.
5. Data collection	The questionnaire was administered to the study population, followed by preliminary evaluation of item distributions, missing data, and floor and ceiling effects.
6. Psychometric validation	Internal consistency, construct validity, convergent and discriminant validity, and structural relationships among constructs were evaluated using Partial Least Squares Structural Equation Modeling (PLS-SEM).
7. Robustness assessment	Sensitivity analyses and exploratory subgroup comparisons were conducted to support interpretation of the findings.

PLS-SEM, partial least squares structural equation modeling. Stages 1–6 are detailed in Section 2.2 and Section 2.3 and stage 7 in Section 2.4.

**Table 2:** Socio-demographic characteristics of the sample (*N* = 504).

Characteristic	Category	n	%
Age	Under 18	63	12.5
	18–25 years	191	37.9
	26–35 years	60	11.9
	36–50 years	95	18.8
	Over 50 years	95	18.8
Gender	Female	357	70.8
	Male	147	29.2
Residence	Urban	356	70.6
	Rural	148	29.4
Education	Lower secondary	8	1.6
	High school	184	36.5
	Bachelor’s degree	197	39.1
	Master’s degree	88	17.5
	Doctorate	27	5.4
Diet type	Omnivore	384	76.2
	Flexitarian	79	15.7
	Gluten-free	20	4.0
	Vegetarian	16	3.2
	Vegan	5	1.0
Net monthly income	Under RON 2000	136	27.0
	RON 2001–3500	103	20.4
	RON 3501–5000	96	19.0
	RON 5001–7000	82	16.3
	Over RON 7000	87	17.3

Percentages are computed on N = 504 and may not sum to 100 owing to rounding. RON, Romanian leu.

**Table 3:** Item descriptive statistics, floor and ceiling effects (N = 504; 1 = strongly disagree, 5 = strongly agree).

Item	Mean	SD	Skewness	Kurtosis	Floor %	Ceiling %
FCM1	3.851	0.937	−0.718	0.491	2.4	26.0
FCM2	3.982	0.953	−1.016	1.062	2.8	32.1
FCM3	3.774	1.029	−0.680	0.018	3.2	26.6
FCM4	3.962	0.981	−0.837	0.344	2.2	34.1
GRV1	3.544	0.982	−0.477	0.109	4.0	16.3
GRV2	3.659	0.992	−0.672	0.402	4.4	19.6
GRV3	3.704	0.938	−0.655	0.537	3.2	19.2
GRV4	3.607	1.044	−0.550	−0.013	4.8	21.0
HCO1	3.823	0.964	−0.817	0.656	3.2	24.8
HCO2	3.857	0.962	−0.894	0.809	3.2	25.8
HCO3	4.030	0.969	−1.072	1.114	3.0	36.1
HCO4	4.073	0.971	−1.115	1.137	2.8	39.1
PNV1	3.349	0.925	−0.067	0.087	3.2	12.1
PNV2	3.532	0.956	−0.386	0.042	3.2	15.5
PNV3	3.520	0.968	−0.326	−0.065	3.2	16.3
PNV4	3.552	0.965	−0.386	0.004	3.2	16.5
WTP1	3.192	1.089	−0.230	−0.532	7.7	11.1
WTP2	3.448	1.104	−0.555	−0.397	6.3	15.3
WTP3	3.411	1.133	−0.477	−0.488	7.3	16.5
PUI1	3.278	1.047	−0.272	−0.301	6.3	12.1
PUI2	3.375	1.036	−0.422	−0.160	6.0	12.9
PUI3	3.278	1.069	−0.287	−0.284	7.3	13.1

Kurtosis is excess kurtosis. Floor/ceiling: percentage selecting the lowest/highest category.

**Table 4:** Measurement model: PLS and CFA standardized loadings, internal-consistency reliability and convergent validity.

	Item	PLS Loading	CFA Loading	α	ρc	AVE
Food Choice Motives (FCM)	FCM1	0.882	0.835	0.914	0.940	0.796
	FCM2	0.914	0.890			
	FCM3	0.863	0.806			
	FCM4	0.910	0.887			
Green Values (GRV)	GRV1	0.897	0.871	0.911	0.938	0.790
	GRV2	0.835	0.755			
	GRV3	0.909	0.876			
	GRV4	0.913	0.895			
Health Consciousness (HCO)	HCO1	0.905	0.871	0.938	0.956	0.844
	HCO2	0.934	0.916			
	HCO3	0.928	0.902			
	HCO4	0.908	0.872			
Perceived Nutritional Value (PNV)	PNV1	0.864	0.796	0.931	0.951	0.830
	PNV2	0.934	0.914			
	PNV3	0.929	0.920			
	PNV4	0.915	0.887			
Willingness to Pay (WTP)	WTP1	0.910	0.846	0.898	0.936	0.830
	WTP2	0.928	0.913			
	WTP3	0.895	0.834			
Purchase Intention (PUI)	PUI1	0.938	0.899	0.937	0.960	0.889
	PUI2	0.951	0.934			
	PUI3	0.939	0.906			

All loadings significant at *p* < 0.001 (5000-resample bootstrap/CFA maximum likelihood). ρc, composite reliability; AVE, average variance extracted.

**Table 5:** Discriminant validity: Fornell–Larcker criterion and HTMT ratios.

Construct	FCM	GRV	HCO	PNV	WTP	PUI
FCM	**0.892**	0.611	0.714	0.488	0.450	0.368
GRV	0.666	**0.889**	0.633	0.575	0.489	0.443
HCO	0.768	0.682	**0.919**	0.443	0.428	0.323
PNV	0.527	0.624	0.473	**0.911**	0.549	0.543
WTP	0.499	0.540	0.467	0.600	**0.911**	0.639
PUI	0.396	0.480	0.344	0.581	0.691	**0.943**

Diagonal (bold), √AVE; above diagonal, latent-variable correlations; below diagonal, HTMT ratios.

**Table 6:** Test–retest reliability over a 14-day interval (n = 30).

Construct	M (T1)	M (T2)	Pearson r	ICC(2,1)	95% CI	Interpretation
Food Choice Motives	4.22	4.13	0.884	0.877	[0.76, 0.94]	Good
Green Values	3.87	3.92	0.830	0.823	[0.66, 0.91]	Good
Health Consciousness	4.32	4.32	0.834	0.838	[0.69, 0.92]	Good
Perceived Nutritional Value	3.93	4.03	0.914	0.909	[0.82, 0.96]	Excellent
Willingness to Pay	3.91	3.93	0.876	0.866	[0.74, 0.93]	Good
Purchase Intention	3.56	3.60	0.841	0.844	[0.70, 0.92]	Good

ICC(2,1), two-way mixed-effects, absolute-agreement, single-measure intraclass correlation coefficient; CI, confidence interval. ICC values were interpreted according to the criteria of Koo and Li [25]. The interpretation thresholds are reported in Section 2.4. All Pearson correlation coefficients were statistically significant (*p* < 0.001).

**Table 7:** Structural model: direct effects (H1–H7), 5000-resample bootstrapping.

Hypothesis Testing	Path	β	SE	t	*p*-Value	95% CI	f^2^	Decision
H1	FCM → HCO	0.523	0.047	11.18	<0.001	[0.429, 0.614]	0.400	Supported
H2	GRV → HCO	0.314	0.050	6.29		[0.216, 0.412]	0.144	Supported
H3	FCM → PNV	0.218	0.050	4.35		[0.120, 0.315]	0.047	Supported
H4	GRV → PNV	0.442	0.048	9.26		[0.347, 0.533]	0.192	Supported
H5	PNV → WTP	0.549	0.042	13.17		[0.468, 0.629]	0.432	Supported
H6	PNV → PUI	0.275	0.056	4.95		[0.163, 0.381]	0.098	Supported
H7	WTP → PUI	0.488	0.049	10.00		[0.392, 0.582]	0.308	Supported

β, standardised path coefficient; f^2^, effect size (interpretation thresholds are reported in Section 2.4). Endogenous R^2^: HCO 0.572; PNV 0.361; WTP 0.302; PUI 0.461. Q^2^: 0.477/0.292/0.247/0.395. Inner VIF 1.000–1.596.

**Table 8:** Mediation analysis: specific indirect effects (H8–H10), 5000-resample bootstrapping.

Hypothesis Testing	Indirect Path	Effect	SE	t	*p*-Value	95% CI	Mediation Type	Decision
H8	FCM → PNV → PUI	0.060	0.019	3.17	0.002	[0.026, 0.101]	Indirect-only	Supported
H9	GRV → PNV → PUI	0.122	0.028	4.34	<0.001	[0.068, 0.177]	Indirect-only	Supported
H10	PNV → WTP → PUI	0.268	0.037	7.21		[0.199, 0.344]	Complementary	Supported

Total effect PNV → PUI = 0.543; the proportion of this total effect accounted for by the indirect path PNV → WTP → PUI (H10) was approximately 49%.

**Table 9:** Sensitivity analysis: reliability and convergent validity for the full sample (N = 504) versus adults only (N = 441).

Construct	α (N = 504)	α (N = 441)	AVE (N = 504)	AVE (N = 441)
FCM	0.914	0.913	0.796	0.795
GRV	0.911	0.915	0.790	0.798
HCO	0.938	0.940	0.844	0.847
PNV	0.931	0.936	0.830	0.840
WTP	0.898	0.897	0.830	0.830
PUI	0.937	0.939	0.889	0.892

Structural results (N = 441): R^2^_PUI = 0.464; all paths *p* < 0.001; all ten hypotheses supported.

## Data Availability

The de-identified dataset supporting the conclusions of this article is available from the correspondence authors upon reasonable request. The complete dataset is not publicly available because it is reserved for additional planned analyses within the ongoing research project.

## Supplementary Materials

The following supporting information can be downloaded at: https://www.mdpi.com/article/10.3390/foods15172950/s1, Supplementary Table S1: Complete item list for the structural model: construct, item code, original (Romanian) wording, English translation, source scale, and final standardised outer loading (N = 504); Supplementary Table S2: Constructs measured in the full questionnaire but excluded from the structural model; Supplementary File S1: Clean questionnaire (English version).

## Abbreviations

The following abbreviations are used in this manuscript:

α	Cronbach’s alpha
AVE	Average variance extracted
CFA	Confirmatory factor analysis
CFI	Comparative fit index
CI	Confidence interval
CR	Composite reliability
EFA	Exploratory factor analysis
FCM	Food choice motives
GRV	Green values
HCO	Health consciousness
HTMT	Heterotrait–monotrait ratio
ICC	Intraclass correlation coefficient
KMO	Kaiser–Meyer–Olkin measure
NFI	Normed Fit Index
PLS-SEM	Partial Least Squares Structural Equation Modeling
PNV	Perceived nutritional value
PUI	Purchase intention
Q^2^	Predictive relevance
RMSEA	Root Mean Square Error of Approximation
R^2^	Coefficient of determination
SD	Standard deviation
SRMR	Standardized Root Mean Square Residual
TLI	Tucker–Lewis index
VIF	Variance inflation factor
WTP	Willingness to pay
