# Supplementary material for: Development and Validation of a Multidimensional Consumer Assessment Framework for Alternative-Ingredient Bakery and Confectionery Products: Implications for Nutrition Methodology and Sustainable Diet Research
Source: Foods. 2026 Aug 22;15(17):2950. doi: 10.3390/foods15172950 (PMC13565372; doi:10.3390/foods15172950)
Supplement: Supplementary file 1 [file foods-15-02950-s001.zip › Supplementary File S1.pdf]

Supplementary File S1. Clean questionnaire (English version)

Title: Consumer perceptions of alternative raw materials in pastry/confectionery and their connection to healthy and sustainable diets.

Purpose: To identify perceptions, preferences, barriers and consumption intentions regarding the acceptance of alternative ingredients (e.g., wholegrain/gluten-free flours, natural sweeteners, unrefined plant-based fats) in connection with healthy and sustainable diets.

Confidentiality: Responses are anonymous and used strictly for academic purposes. **Estimated time:** 5–8 minutes.

Instructions: For each statement, please indicate your level of agreement on the following scale: **1 = Strongly disagree; 2 = Disagree; 3 = Neither agree nor disagree; 4 = Agree; 5 = Strongly agree.**

Section S1 — Health Consciousness (modelled: HCO)

1.1 I am very attentive to how healthy the foods I eat are.

1.2 I choose foods carefully in order to maintain my health.

1.3 I consider health an important criterion when choosing food products.

1.4 I am concerned about the impact of diet on my state of health.

Section S2 — Green / Sustainable Consumption Values (modelled: GRV; 4 of 6 items retained)

2.1 I am willing to make personal compromises to protect the environment. *(GRV2)*

2.2 I prefer products that are less harmful to the environment. *(not retained)*

2.3 I try to buy products that respect sustainability principles. *(GRV3)*

2.4 Choosing eco-friendly products is important to me. *(not retained)*

2.5 The environmental impact influences my purchasing decisions. *(GRV1)*

2.6 I feel responsible for protecting the environment through my consumption choices. *(GRV4)*

Section S3 — Perceived Nutritional Value (modelled: PNV; custom scale)

3.1 Products made with alternative raw materials have a higher nutritional value.

3.2 These products have a better nutritional profile (e.g., more fibre, fewer refined ingredients).

3.3 These products are nutritionally more suitable for regular consumption.

3.4 These products provide real nutritional benefits, not just a "healthier" image.

Section S4 — New Environmental Paradigm (NEP, short) (measured; not in structural model)

4.1 People must live in harmony with nature in order to survive.

4.2 Human intervention in nature often has negative consequences.

4.3 The Earth's natural resources are limited.

Section S5 — Food Choice Motives (FCQ – Health & Natural Content) (modelled: FCM)

5.1 I choose foods that are good for my health.

5.2 I prefer foods that contain natural ingredients.

5.3 I avoid foods with artificial or highly processed ingredients.

5.4 It is important to me that foods are as natural as possible.

Section S6 — Food Neophobia (measured; not in structural model; (R) = reverse-scored at analysis)

6.1 I am reluctant to try foods I have not eaten before. (R)

6.2 I like to try new and different foods.

6.3 I am curious to taste unfamiliar food products.

6.4 I prefer to eat only foods I am already familiar with. (R)

Section S7 — Frequency of Consumption (last 30 days) (measured; not in structural model)

7.1 In the last 30 days, I consumed pastry/confectionery products made with alternative raw materials.

7.2 In the last 30 days, I chose these products instead of conventional ones.

7.3 In the last 30 days, I frequently bought products with alternative ingredients.

7.4 In the last 30 days, these products were a regular part of my diet.

Section S8 — Trust in Food Labelling / Information (measured; not in structural model)

8.1 I trust the information on food product labels.

8.2 I am sceptical about the nutritional claims of food products. (R)

8.3 I consider that information about ingredients and benefits is generally credible.

Section S9 — Willingness to Pay a Premium (modelled: WTP)

9.1 I am willing to pay more for pastry/confectionery products made with alternative raw materials.

9.2 I would accept a higher price for such products if they are healthier.

9.3 A higher price is justified for products with superior nutritional benefits.

Section S10 — Purchase Intention (modelled: PUI)

10.1 I intend to buy pastry/confectionery products made with alternative raw materials.

10.2 It is likely that I will choose these products in the future.

10.3 I will prefer these products over conventional ones.

Section S11 — Word-of-Mouth Intention (measured; not in structural model)

11.1 I would recommend pastry/confectionery products made with alternative raw materials to others.

11.2 I would speak positively about these products to friends or family.

Demographic block

- **Age:** Under 18 / 18–25 / 26–35 / 36–50 / Over 50
- **Residence:** Urban / Rural
- **Gender:** Female / Male / Prefer not to say
- **Education:** Lower secondary (gymnasium) / High school / Bachelor's / Master's / Doctorate
- **Diet type:** Omnivore / Flexitarian / Vegetarian / Vegan / Gluten-free
- **Net personal monthly income (optional):** Under RON 2,000 / RON 2,001–3,500 / RON 3,501–5,000 / RON 5,001–7,000 / Over RON 7,000 / Prefer not to say
