# Supplementary material for: Development and Validation of a Multidimensional Consumer Assessment Framework for Alternative-Ingredient Bakery and Confectionery Products: Implications for Nutrition Methodology and Sustainable Diet Research
Source: Foods. 2026 Aug 22;15(17):2950. doi: 10.3390/foods15172950 (PMC13565372; doi:10.3390/foods15172950)
Supplement: Supplementary file 1 [file foods-15-02950-s001.zip › Supplementary Table S1.pdf]

Supplementary Table S1. Complete item list for the structural model: construct, item code, original (Romanian) wording, English translation, source scale, and final standardised outer loading (N = 504)

Construct	Code	Original item (Romanian, as administered)	English translation	Source / theoretical basis	Loading
Food Choice Motives (FCM)	FCM1	Aleg alimente care sunt benefice pentru sănătatea mea.	I choose foods that are good for my health.	Food Choice Questionnaire – health dimension; Steptoe, Pollard & Wardle (1995) [15]	0.882
	FCM2	Prefer alimente care conțin ingrediente naturale.	I prefer foods that contain natural ingredients.	FCQ – natural-content dimension; Steptoe et al. (1995) [15]	0.914
	FCM3	Evit alimentele cu ingrediente artificiale sau foarte procesate.	I avoid foods with artificial or highly processed ingredients.	FCQ – natural-content dimension; Steptoe et al. (1995) [15]	0.863
	FCM4	Este important pentru mine ca alimentele să fie cât mai naturale.	It is important to me that foods are as natural as possible.	FCQ – natural-content dimension; Steptoe et al. (1995) [15]	0.910
Green Values (GRV)	GRV1	Impactul asupra mediului influențează deciziile mele de cumpărare.	The environmental impact influences my purchasing decisions.	GREEN consumption-values scale; Haws, Winterich & Naylor (2014) [13]	0.897
	GRV2	Sunt dispus(ă) să fac compromisuri personale pentru a proteja mediul.	I am willing to make personal compromises to protect the environment.	GREEN scale; Haws et al. (2014) [13]	0.835
	GRV3	Încerc să cumpăr produse care respectă principiile sustenabilității.	I try to buy products that respect sustainability principles.	GREEN scale; Haws et al. (2014) [13]	0.909
	GRV4	Mă simt responsabil(ă) pentru protejarea mediului prin alegerile mele de consum.	I feel responsible for protecting the environment through my consumption choices.	GREEN scale; Haws et al. (2014) [13]	0.913
Health Consciousness (HCO)	HCO1	Sunt foarte atent(ă) la cât de sănătoase sunt alimentele pe care le consum.	I am very attentive to how healthy the foods I eat are.	Health Consciousness Scale; Gould (1990) [14]	0.905
	HCO2	Aleg alimentele cu grijă pentru a-mi menține sănătatea.	I choose foods carefully in order to maintain my health.	HCS; Gould (1990) [14]	0.934
	HCO3	Consider sănătatea un criteriu important atunci când aleg produsele alimentare.	I consider health an important criterion when choosing food products.	HCS; Gould (1990) [14]	0.928
	HCO4	Mă preocupă impactul alimentației asupra stării mele de sănătate.	I am concerned about the impact of diet on my state of health.	HCS; Gould (1990) [14]	0.908

Perceived Nutritional Value (PNV)	PN V1	Produsele realizate cu materii prime alternative au o valoare nutrițională mai ridicată.	Products made with alternative raw materials have a higher nutritional value.	Custom scale developed for this study (food-reformulation & nutrition-perception literature)	0.864
	PN V2	Aceste produse au un profil nutrițional mai bun (ex. mai multe fibre, mai puține ingrediente rafinate).	These products have a better nutritional profile (e.g., more fibre, fewer refined ingredients).	Custom scale developed for this study	0.934
	PN V3	Aceste produse sunt mai adecvate din punct de vedere nutrițional pentru consum regulat.	These products are nutritionally more suitable for regular consumption.	Custom scale developed for this study	0.929
	PN V4	Aceste produse oferă beneficii nutriționale reale, nu doar o imagine de produs „mai sănătos".	These products provide real nutritional benefits, not just a "healthier" image.	Custom scale developed for this study	0.915
Willingness to Pay (WTP)	WT P1	Sunt dispus(ă) să plătesc mai mult pentru produse de patiserie/cofetărie cu materii prime alternative.	I am willing to pay more for pastry/confectionery products made with alternative raw materials.	Willingness-to-pay / premium-price acceptance; adapted from Haws et al. (2014) [13]; Laroche et al. (2001) [19]	0.910
	WT P2	Aș accepta un preț mai ridicat pentru astfel de produse dacă sunt mai sănătoase.	I would accept a higher price for such products if they are healthier.	WTP literature; Laroche et al. (2001) [19]	0.928
	WT P3	Prețul mai mare este justificat pentru produsele cu beneficii nutriționale superioare.	A higher price is justified for products with superior nutritional benefits.	WTP literature; Dodds et al. (1991) [18]; Laroche et al. (2001) [19]	0.895
Purchase Intention (PUI)	PU I1	Intenționez să cumpăr produse de patiserie/cofetărie realizate cu materii prime alternative.	I intend to buy pastry/confectionery products made with alternative raw materials.	Purchase Intention; Ajzen (1991) [17]; Dodds et al. (1991) [18]	0.938
	PU I2	Este probabil să aleg aceste produse în viitor.	It is likely that I will choose these products in the future.	Purchase Intention; Ajzen (1991) [17]; Dodds et al. (1991) [18]	0.951
	PU I3	Voi prefera aceste produse în detrimentul celor convenționale.	I will prefer these products over conventional ones.	Purchase Intention; Ajzen (1991) [17]; Dodds et al. (1991) [18]	0.939

All items used a five-point Likert agreement scale (1 = strongly disagree … 5 = strongly agree). Loadings are standardised outer loadings from the consistent PLS (Mode A) estimation, N = 504; all significant at p < 0.001 (5,000-resample bootstrap.)

Notes on item selection.

- *Green Values:* the administered questionnaire contained six sustainability items; the four items above were retained for the validated scale on the basis of conceptual correspondence to the GREEN value domain and

item-total coherence. The two items not retained were *″Prefer produse care sunt mai puțin dăunătoare pentru mediu″* ("I prefer products that are less harmful to the environment") and *″Alegerea produselor ecologice este importantă pentru mine″* ("Choosing eco-friendly products is important to me").

- *Food Choice Motives:* the administered items operationalise the **health** and **natural-content** dimensions of the FCQ only (see author note in the main text, Section 2.2).
