# Supplementary material for: Development and Validation of a Multidimensional Consumer Assessment Framework for Alternative-Ingredient Bakery and Confectionery Products: Implications for Nutrition Methodology and Sustainable Diet Research
Source: Foods. 2026 Aug 22;15(17):2950. doi: 10.3390/foods15172950 (PMC13565372; doi:10.3390/foods15172950)
Supplement: Supplementary file 1 [file foods-15-02950-s001.zip › Supplementary Table S2.pdf]

Supplementary Table S2. Constructs measured in the full questionnaire but excluded from the structural model

Domain	Items	Reason for exclusion
New Environmental Paradigm (NEP, short)	3	Conceptual overlap with the retained Green Values construct
Food Neophobia Scale (FNS)	4	Measured for descriptive/exploratory purposes; outside the core value→evaluation→intention chain; contains reverse-scored items
Frequency of Consumption (last 30 days)	4	Behavioural/formative-leaning indicator, not a reflective attitudinal construct
Trust in Food Labelling / Information	3	Measured for descriptive purposes; not part of the hypothesised model; contains a reverse-scored item
Word-of-Mouth Intention (WOM)	2	Only two items — below the threshold recommended for stable reflective SEM estimation

These domains were retained in the questionnaire for descriptive and future exploratory analyses but were excluded a priori from the structural model on grounds of parsimony and preliminary measurement adequacy (main text, Section 2.2).
